# Immune Responses to Plague Infection in Wild *Rattus rattus*, in Madagascar: A Role in Foci Persistence?

**DOI:** 10.1371/journal.pone.0038630

**Published:** 2012-06-18

**Authors:** Voahangy Andrianaivoarimanana, Sandra Telfer, Minoarisoa Rajerison, Michel A. Ranjalahy, Fehivola Andriamiarimanana, Corinne Rahaingosoamamitiana, Lila Rahalison, Ronan Jambou

**Affiliations:** 1 Unité Peste, Institut Pasteur de Madagascar, Antananarivo, Madagascar; 2 School of Biological Sciences, University of Aberdeen, Aberdeen, United Kingdom; 3 Unité d’Immunologie, Institut Pasteur de Madagascar, Antananarivo, Madagascar; University of California Merced, United States of America

## Abstract

**Background:**

Plague is endemic within the central highlands of Madagascar, where its main reservoir is the black rat, *Rattus rattus*. Typically this species is considered susceptible to plague, rapidly dying after infection inducing the spread of infected fleas and, therefore, dissemination of the disease to humans. However, persistence of transmission foci in the same area from year to year, supposes mechanisms of maintenance among which rat immune responses could play a major role. Immunity against plague and subsequent rat survival could play an important role in the stabilization of the foci. In this study, we aimed to investigate serological responses to plague in wild black rats from endemic areas of Madagascar. In addition, we evaluate the use of a recently developed rapid serological diagnostic test to investigate the immune response of potential reservoir hosts in plague foci.

**Methodology/Principal Findings:**

We experimentally infected wild rats with *Yersinia pestis* to investigate short and long-term antibody responses. Anti-F1 IgM and IgG were detected to evaluate this antibody response. High levels of anti-F1 IgM and IgG were found in rats one and three weeks respectively after challenge, with responses greatly differing between villages. Plateau in anti-F1 IgM and IgG responses were reached for as few as 500 and 1500 colony forming units (cfu) inoculated respectively. More than 10% of rats were able to maintain anti-F1 responses for more than one year. This anti-F1 response was conveniently followed using dipsticks.

**Conclusion/Significance:**

Inoculation of very few bacteria is sufficient to induce high immune response in wild rats, allowing their survival after infection. A great heterogeneity of rat immune responses was found within and between villages which could heavily impact on plague epidemiology. In addition, results indicate that, in the field, anti-F1 dipsticks are efficient to investigate plague outbreaks several months after transmission.

## Introduction

Plague is a zoonotic disease, caused by *Yersinia pestis,* and transmitted from small rodents to humans by bites from infected fleas. In humans, plague infection can remain localized in lymph nodes or develop into a fatal lung infection [1]. An average of 2,300 human cases of plague and 150 deaths are recorded annually. More than 96% of all cases and deaths are currently reported from Africa, with a quarter of them occurring in Madagascar [2]. During the 1990s, reappearance of plague in several countries demonstrates that it can be considered as a re-emerging disease [3], [4]. Introduced in 1898 to Madagascar by steamboats from India [3], plague has become endemic in the central highlands at altitudes above 800 meters. In rural areas the black rat, *R. rattus*, is the most abundant small mammal, usually accounting for more than 85% of small mammals captured, whilst other species such as *Mus musculus* and *Suncus murinus* are trapped much less frequently [5]. Thus, even though *R. rattus* is reputedly sensitive to plague infection, it appears that this species is the key reservoir host for plague in these areas. A number of factors may explain plague persistence in such a system, including spatial structure within host populations resulting in non-synchronous epidemics [6] and/or host phenotypes that show resistance against the bacteria [7]. In Madagascar at least some black rats from the endemic plague zone appear to have evolved resistance [8] with this resistance linked to genetic factors [9], [10]. Although laboratory mice and rats have been widely used to study immune responses against plague, and persistence of antibodies up to 8 months after experimental immunization have been reported [11], [12], immune responses have been poorly investigated in natural hosts of the bacteria, including wild *R. rattus* from Madagascar [8], [13], [14], [15].

Studies demonstrate that F1, V antigen, YopH, YopM, YopD, and Pla are major antigens recognized by mice after infection [16]. F1 is a capsular antigen expressed in fleas with an anti-phagocytic activity [17]. It is essential for virulence after flea bite [18] but not for plague pathogenesis [19]. However anti- F1 titers are predictive of protection against *Y. pestis* [20], [21], even if they don’t truly correlate with it [22]. *Y. pestis* F1 antigen is thus widely investigated as a vaccine candidate and is the basis of a rapid diagnostic test (RDT) of infection [23]. Detection of anti-F1 plague antibodies is also used to confirm plague diagnosis, and, following earlier works [24], [25], we recently described a new RDT to detect both IgM and IgG antibodies in humans and animals [26].

Retrospective serological investigations of antibodies against F1 in wild rodents have also been an important strategy to investigate foci, including Madagascar [4], [5]. However, a lack of understanding of immune response kinetics in wild rats complicates interpretations of the results and explorations of the role of immune responses in plague epidemiology.

To investigate the role that black rat immune responses may play in plague persistence in Madagascar and facilitate future serological investigations of reservoir hosts in Madagascar and elsewhere, we (i) analyzed anti-F1 IgM and IgG responses in wild rats challenged with different doses of *Y. pestis*, (ii) examined antibody persistence and (iii) evaluated the use of the anti-F1 RDT as a tool for investigating plague foci.

## Methods

### Animal Sampling

Rats were collected from small villages around Betafo, a major plague focus of the Highlands. Trapping methodologies followed standard protocols [5]. The sex of each rat was determined, and fleas were collected by brushing. To ensure any remaining fleas were killed, rats were transported back to the laboratory in cages treated with insecticide. Rats were kept for 2 weeks for observation. Presence of anti-F1 antibodies was checked for each rat and only seronegative individuals were included in the study. For the four villages sampled 425 rats were captured and only 1.6% of them were anti-F1 positive. Animals were not checked for previous *Yersinia enterocolitica*, *Yersinia pseudotuberculosis* or F1-negative *Y. pestis* infection. However, as previously published these strains have never been described in Madagascar [27]. Although, no national committee is yet organized in Madagascar, all experimental protocols were reviewed and validated by our Institutional Ad hoc committee for the care and use of animals. The study has been conducted in accordance with the Institut Pasteur guidelines (http://www.pasteur.fr/ip/easysite/pasteur/en/institut-pasteur/ethics-charter) for animal husbandry and experiments which adheres to the French animal ethic chart (CNRS, Paris). All experiments were performed at Biosafety level 2. Householders gave their informed consent for sampling rats in the household.

### Experimental Plague Challenge

For the short term follow-up of the immune response (up to one month), we used i) a group of 118 rats caught in two villages (Ambohimasina and Maromanana), on which both anti-F1 IgM and IgG were measured, and ii) a further group of 88 rats collected in two other villages in the same area (Andratsaimahamasina and Malaza) on which, due to logistical limitations, only anti-F1 IgG were measured. Four males and four females from each village were randomly assigned to each dose group and were inoculated with 15, 150, 1,500, 15,000 or 30,000 colony-forming-units (cfu) of *Y. pestis* (F1 positive 40/09B strain was isolated from bubo aspirate of a Malagasy patient and routinely maintained in the laboratory as reference strain). Each dose of *Y. pestis* was delivered subcutaneously into the left thigh, an administration route that closely resembles the bite of an infective flea. The negative control group received only sterile Brain Heart Infusion (BHI). Rats were housed in polycarbonate cages of two rats each, with filter tops in a ventilated cabinet (TECHNIPLAST), at ambient temperature, with food and water *ad libitum*. They were examined four times daily. At the end of each experiment or upon signs of terminal disease, rats were euthanized using carbon dioxide (Charles River Laboratories). An antigen F1 RDT and bacteriology culture were carried out on the spleen of dead rats to confirm that death was due to plague.

Blood samples were collected on seropads (LDA Zoopole- B.P.54, 22440 Ploufragan) and into dry tubes at Day 0, Day 8, Day 13, Day 18 and Day 25. In addition, full blood counts were done (FBC) using Kova slides (HYCOR Biomedical).

For the long term follow up of antibody, a third group of 84 rats was caught in three other villages (Ambohimanana, Amparihimboahangy and Belanitra). The group was composed of 48 males and 36 females, captured from sisal hedges (n = 42), houses (n = 26) and rice fields (n = 16). They were housed in group boxes of three rats each. Their average weight was 119.4±2 g before plague inoculation. They were all inoculated with 125 cfu of 23/07S (a F1 positive strain with the same virulence as 40/09B, also maintained as reference strain in the laboratory) and blood sampled every month. Only anti-F1 IgG was measured in this group. As the lifespan of wild rats is typically less than a year, we stopped the experiment after 12.5 months.

### Detection of Anti-F1 Antibodies

Detection of anti-F1 IgG was conducted by enzyme-linked immunosorbent assay (ELISA) as previously described [28] with modifications. When seropads were used, they were soaked overnight in 400 µL of PBS- 0.05% tween- 5% skim milk at 4°C and the resulting liquid was used for ELISA without further dilution. An anti-rat IgG peroxidase conjugate (Sigma, 1∶10000) was used for the revelation step. The mean optical density (OD) obtained against the coating buffer alone was subtracted from the OD against F1 antigen (delta OD).

For anti-F1 IgM detection, a sandwich ELISA was standardized on sera. Briefly, microwells (Maxisorp Nunc-immuno) were coated overnight at 4°C with goat anti-rat IgM (Sera-lab 1 µg/mL) in carbonate buffer pH 9.6. After washing, plates were blocked with PBS- 0.05% tween- 5% skim milk for 2 hours at 37°C and washed again. Rat sera were diluted 1∶200 in PBS- 0.1% tween- 0.5% skim milk and incubated for 1 hour in duplicate. F1-antigen was applied in PBS- 0.1% tween- 0.5% skim milk at 5 µg/mL and incubated overnight at 4°C. After washing, an anti-F1 rabbit polyclonal serum was added (1 µg/mL) for one hour at 37°C and detected with an anti-rabbit IgG peroxidase conjugate (Sigma, 1∶2000).

In each plate, negative and positive sera from wild rodents were included as controls. To allow quantitative inter-plate and inter-experiment comparisons and kinetic analysis of the data, sera were randomly scattered on the ELISA plates. For short term kinetic analysis we expressed results as the ratio (R) of the delta OD of the sample to the mean delta OD of negative sera +3 Standard Deviations (OD ratio). For IgM and IgG, samples with an OD ratio≥2 were considered as positive. For graphic representation, all negative samples were set to zero. For IgG the threshold of positivity was set at 0.05 when using seropads.

### Comparison of ELISA and Dipstick Detection of Anti-F1 IgG

Comparison of the two methods was carried out using whole blood collected on seropads obtained from experimental plague challenged rats, as these are the samples usually collected in the field. ELISA detection was considered as the reference method and expressed as delta OD. Seropads were grouped by class according to their delta OD from 0.050 to 0.800. Ten samples were considered for analysis for each interval of delta OD, except for interval “>0.800” for which only 7 samples were available. RDT results were scored positive when two pink lines appeared and negative when only one pink line was observed [23], [26]. For each range of delta OD, we determined i) the percent of samples scored as positive with dipstick and ii) the cumulative percent of samples with delta OD less than this range and detected as positive by dipstick.

Using the group of rats followed for one year, we then applied these results to evaluate the ability of the RDT to detect positive rats in field populations by assessing how the RDT would be expected to perform at different time points after infection. At each sampling date we sorted the samples according to their delta OD into the classes described above, and for each class we predicted the number of samples expected to score positive using the RDT. We plotted this predicted percent positive by RDT and the true percent positive by ELISA.

### Statistical Analysis

To analyze the long term IgG responses, we defined for each rat: the maximal OD ratio (ODmax), the time to reach this maximum, the time to observe decay by half of the ODmax (T1/2), the time to switch from positive to negative and the terminal OD ratio at the end of follow up or at death. In the long-term follow-up, the persistence of IgG antibodies was followed until rats had two consecutive negative detections of antibodies. Statistical analysis was performed on Statistica v7. Intergroup analysis was performed using Mann Whitney and Kruskal Wallis tests. Non Parametric correlations were calculated using Spearman test. Linear multiple regressions were used to evaluate multiparameter effects on OD ratio. Only additive effects were considered due to the relatively small sample size per dose.

## Results

### Short- term Kinetics of Anti-F1 IgM and IgG Antibody Responses after Y. pestis Challenge

The rats from Ambohimasina and Maromanana harbored an average of 1.6±2 fleas, had a leukocyte count of 11050±5330 and an erythrocyte count of 10.4±4.5 million/µL. A significant difference in weight (p = 0.002 MW) was found at capture time between the two villages. No other differences were detected. Although rats were randomly allocated to dose (see methods), a significant higher count of leukocytes was found at Day 0 for the group 1,500, 15,000 and 30,000 cfu in comparison with the control group (MW p = 0.007 for each).

Among the 118 rats, 28 died before Day 10 after inoculation (none in the control group). No significant difference was found between dead rats and surviving ones for weight, erythrocyte or leukocyte counts, sex or number of fleas. Mortality increases with dose from 18% to 37%. The time needed to kill half of the rats decreased from Day 6 to Day 4.

**Figure 1:**
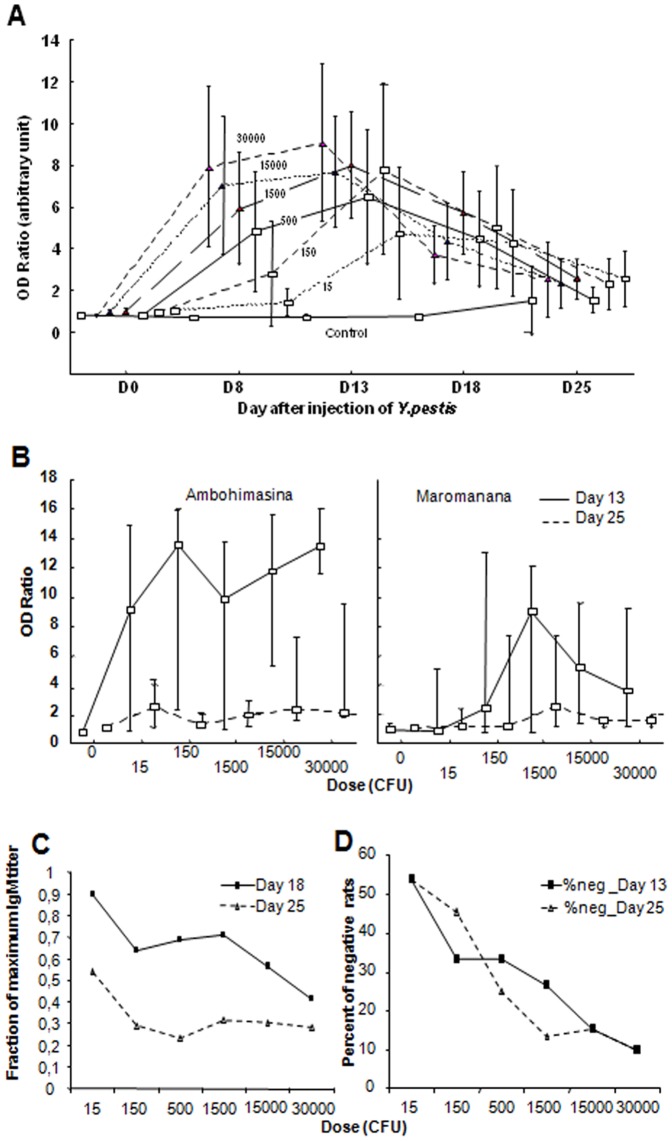
Short term kinetic of anti-F1 IgM in *R. rattus* after inoculation with *Y. pestis*. Wild healthy rats (*Rattus rattus*) were inoculated with different doses of living *Y. pestis* (0 to 30,000 cfu) and blood sampled over one month. Anti-F1 IgM was detected by a sandwich ELISA. Results were expressed as the ratio of the mean optical density (OD) of the sample on the mean OD obtained for negative control rats +3 SD. Rats are from two villages Ambohimasina and Maromanana. (median; bar: 10% and 90% percentiles) A/Time course of the median OD ratio of each group: OD ratio peaked at Day 13 and decreased rapidly after B/Median of OD ratio obtained at Day13 and Day 25 for the two villages according to the dose inoculated. Rats from Ambohimasina produced a stronger IgM response against *Y. pestis* than Maromanana rats C/Median fraction of maximal anti-F1 IgM titer (OD ratio, mostly at Day 13) remaining at Day 18 and Day 25 according to the dose inoculated. For each rat the maximal OD ratio is noted as well as the OD ratio at Day 18 and Day 25, the fractions are calculated and the median of each group is plotted according to the dose inoculated. The speed of decay of IgM increases with the dose inoculated. D/Percent of rats remaining seronegative after inoculation. For each dose the fraction of rats remaining negative for anti-F1 IgM at Day 13 and IgG at Day 25 is plotted. It decreases from 15 to 30,000 cfu.

Whatever the dose inoculated, anti-F1 IgM appeared between Day 0 and Day 8, increased up to Day 13, and then declined again (Figure 1A). Depending on the dose, the mean OD ratio was between 4 and 9 at Day 13, and decreased to an average of 2 at Day 25 (for all doses). This decay was faster for higher inoculation doses than for low ones (Figure 1C) The percentage of rats staying negative despite inoculation also decreased with dose from 50% (15 cfu) to 10% (30,000 cfu) (Figure 1D). No significant association was found between the OD ratio at Day13 and parameters of the rat itself at inoculation (weight, erythrocyte and leukocyte count, sex, fleas). However at Day 13, a strong effect of village was found (Figure 1B). To induce IgM maximal OD, a 100-fold lower dose of bacteria was needed in Ambohimasina (15 cfu) compared to Maromanana (1,500 cfu). This effect was still significant (beta = 0.31, p<0.05) when analyzed in multiple regression with the dose and the parameters of the rat (Table 1) as confounding variables.

**Table 1:** Multiple regression analysis of the anti-F1 IgM and IgG titers.

	(1)	(2)	(3)	(4)
**Explained parameter**	Day 13 anti-F1 IgM		Day 25 anti-F1 IgG	
Nb rats	**82/118**	**65/118**	**84/118**	**67/118**
R Multiple	0,413	0,316	0,329	0,339
F	3,13	1,07	1,89	1,3
R^2^ adjusted (p)	0,116(0,012)	0,007(0,385)	0,051(0,104)	0,026(0,270)
**Beta coefficient**				
Dose	0,151	0,215	**0,274**	**0,313**
Sex (M/F)	0,107	0,021	0,084	0,043
Weight (g)	0,049	−0,13	0,056	−0,02
Nb fleas	−0,12	−0,13	−0,12	−0,04
Erythrocyte count (d0)		−0,04		−0,03
Leukocytes count (d0)		−0,12		0,031
Village	−**0,31**		−0,06	

four separate logistic regressions (1) to (4) were conducted on the set of 118 rats from Ambohimasina and Maromanana studied for short term follow up of IgM and IgG, to check effect of parameters (beta coefficient are reported when parameters are used in the regression and plotted in bold when significant at less than p = 0.05).

**Figure 2:**
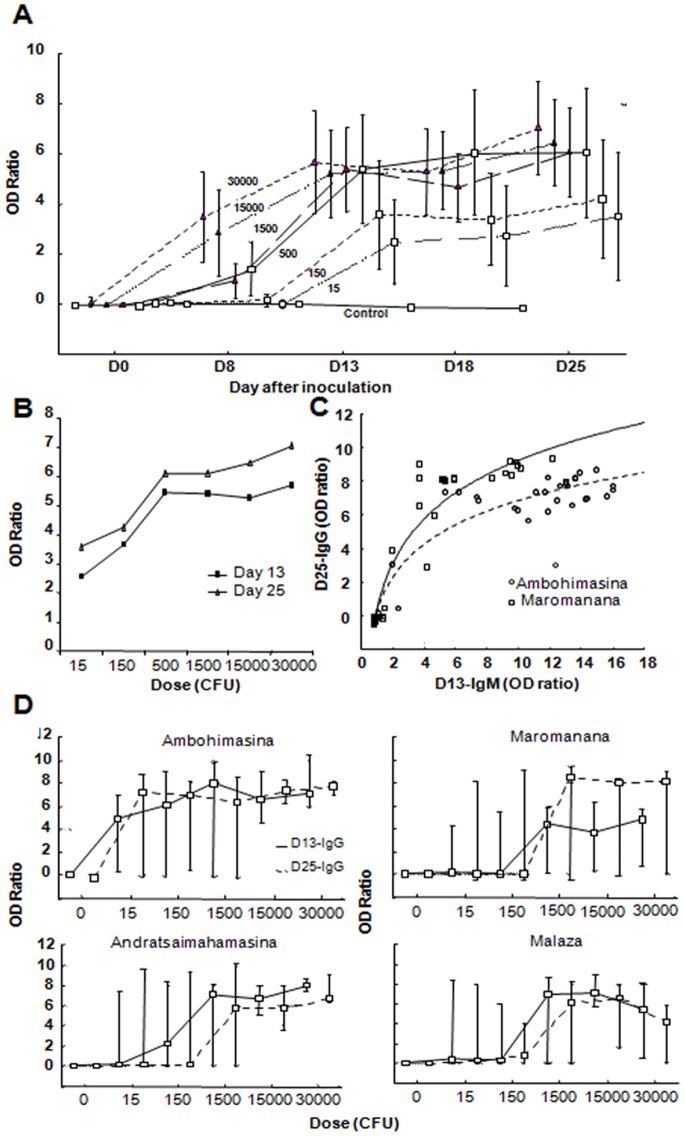
Short term kinetic of anti-F1 IgG in *R. rattus* after inoculation with *Y. pestis.* Wild healthy rats (*Rattus rattus*) were inoculated with different doses of living *Y. pestis* and blood sampled over one month. Anti-F1 IgG was detected by ELISA. Results were expressed as the ratio of the mean optical density (OD) of the sample on the mean OD obtained for negative control rats +3SD. (bar: 10% and 90% percentiles) A/Time course of the median anti-F1 IgG OD ratio of each group: OD ratio peaked between Day 13 and Day 25 and remained stable. Rats are from the two villages Ambohimasina and Maromanana B/Median anti-F1 IgG OD ratio at Day 13 and Day 25 according to the dose of *Y. pestis* inoculated. OD reaches a plateau for 500 cfu. Rats are from the two villages Ambohimasina and Maromanana C/Scatter plot of the OD ratio (IgM at Day 13/IgG at Day25) of rats from Ambohimasina and Maromanana. Logarithmic regression highlighted positive correlations between IgM and IgG responses to *Y. pestis*, (for Maromanana (solid line) Day 25-IgG  = 0,5322+8,7431*log10(Day 13-IgM); for Ambohimasina (dashed line) Day 25-IgG  = 0,4428+6,4026*log10(Day 13-IgM) ). D/Comparison of the dose/anti-F1 IgG titer relation for four different villages. For each group of dose, median OD ratio at Day 13 and Day 25 are plotted. Different sensitivity of the rats according to the village can be shown.

Whatever the dose inoculated, anti-F1 IgG OD ratios reached a plateau after Day 13 (Figure 2A). However, the maximal OD ratio increased with dose up until 500 cfu injected. As with IgM, the percentage of rats staying negative after injection (at Day 25) decreased with dose (Figure 2B). No association was found between the anti-F1 IgG OD ratio (as well as for rats staying negative) and parameters of the rat (sex, erythrocyte and leukocyte count, weight, number of fleas). However, as for IgM, the same “village” effect was observed for the kinetic and maximal OD ratio reached (Figure 2D). However, this effect doesn’t reach significance in multiple regression for which the “dose” remained the major factor modulating titer. Data obtained from Andratsaimahamasina and Malaza indicated that rats from Ambohimasina showed greater IgG responses than the other 3 villages (Figure 2D).

For the first two villages, we also compared for each rat the maximal OD ratio obtained at Day 13 for anti-F1 IgM and at Day 25 for anti-F1 IgG (Figure 2C) and found them positively correlated. However in rats of Ambohimasina where the IgM titers are higher, the IgG titers are lower than Maromanana where IgM are lower. This suggests that high level of IgM could induce an early capture of antigens in blood modulating IgG response.

### Long- term Monitoring of Anti-F1 IgG after *Y. pestis* Experimental Challenge

**Figure 3:**
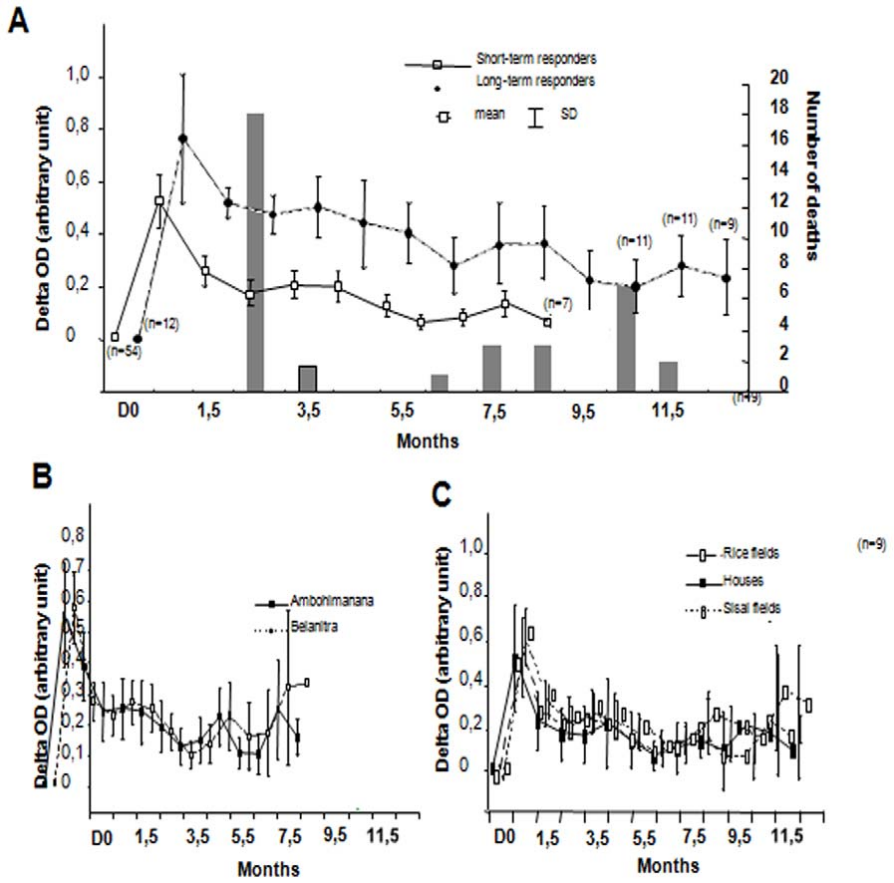
Long term kinetic of anti-F1 IgG in *R. rattus* after inoculation with 125 cfu of *Y. pestis.* Rats captured in three villages were inoculated with 125 cfu of living *Y. pestis* and blood sampled over 12.5 months using seropads. Anti-F1 IgG measured by ELISA delta OD are plotted. A/Mean of delta OD of survival rats were plotted according to time. Columns represent the number of deaths registered at each time point. Data are separated in two groups, i.e. rats with long-lasting IgG response and those with short-lasting response. Almost no death was registered in the long-lasting group. (n: number of remaining rat in the group at time point) B/Same plot but with data separated according to the villages. No difference between villages was found C/Same plot according to the capture site of the rats showing no difference.

During the 18 days following inoculation, 18 rats died without bias in weight, sex or origin (Figure 3A). For the remaining 66 rats, 12 died before one year. For these later deaths, no association was found with sex, weight, flea number and short term anti-F1 IgG kinetic parameters. For all the rats surviving more than one month, no infection was detected at the date of their death suggesting that chronic infection might be unusual.

In concordance with the first experiment, anti-F1 IgG levels rose during the first 2 weeks after *Y. pestis* inoculation. Two groups can be distinguished among the rodents (Figure 3A), i) a minor group (12 out of 66) with long-lasting antibodies (8 of which were still alive and showed persistence of antibodies beyond 12.5 months) and ii) a major group with a negative titer after 8.5 months. For the short-persistence group, delta OD increased to a maximum of 0.6 after 1±1.25 months and decreased rapidly to delta OD less than 0.2 in 6.8±3 months after infection (T1/2 = 3 months). For long-lasting group delta OD increased slower (maximal at 2.5±2 months, not significantly different with previous group, MW). Although the delta OD subsequently declined (T ½  = 4.4 months, not significantly different with previous group, MW), it reached a plateau around 0.4 delta OD and then remained stable until 12.5 months. There was a trend towards lower weight in the long lasting group compared with the short-persistence group (p = 0.07, MW). Weight was also negatively associated with the maximal delta OD, the T1/2 and the time to switch from positive to negative (p = 0.03, 0.018, 0.015 respectively, Spearman). Although females weigh significantly less than males (p = 0.001, MW), sex was not significantly linked to the antibody kinetic parameters used in this study. Thus, as weight is a crude proxy of age in rodents, we believe our results indicate that age may influence the kinetics of immune response to plague. However, it is important to note that relationships between weight and immune responses were not found in the short term kinetic experiment (including the maximal delta OD). We detected no difference in kinetic parameters according to the capture site of the rat (sisal hedges, houses or rice fields) or to the village (Figure 3B, C).

### Comparison of Anti-F1 Detection by Dipstick and ELISA

**Figure 4:**
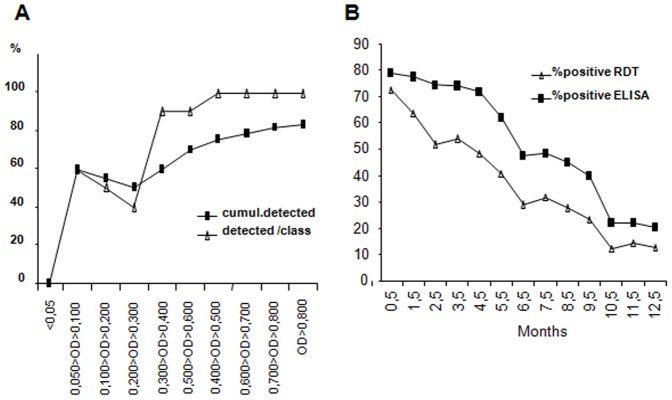
Comparison of detection of anti-F1 IgG by dipstick and ELISA methods. A/Seropads obtained from the long-term follow up are analyzed using both dipstick and ELISA The percent of positive samples by dipstick is plotted according to the delta OD obtained by ELISA. Both percent per class and cumulative percent of positive samples are plotted. B/Comparison of the percent of positive rat detected using ELISA and the expected positive detection using dipsticks. Using the relationship shown in A and the delta OD values represented in Figure 3A, we predicted the percent of positive rats expected using the dipstick method.

Comparison of RDT to the reference test showed that below 0.300 delta OD only an average of 50% of the samples were positive for RDT whereas above 0.300 this concordance increases to 90% (Figure 4A). To compare ELISA and predicted RDT results we used the rats followed over 12 months. Figure 4B plots the percentage of positive rats at different time points according to the method used (actual ELISA results or predicted dipstick results). At Day 18, 52 out of 66 rats were positive (delta OD>0.050, 79%) and 51 out of 66 at 1.5 months (77%). The proportion of rats positive by ELISA starts to decline rapidly after 4.5 months. Based on the delta OD obtained for each rat, we predict the dipstick would have detected 92% (48) and 82% (42) of the positives at Day 18 and 1.5 months respectively. Thus, although the anti-F1 RDT consistently underestimates the prevalence of positive animals, these data confirm the usefulness of the anti-F1 RDT to explore plague epidemics in the field.

## Discussion

To study the kinetic of the serological immune response of wild *R. rattus* against *Y. pestis*, rats were collected in the field, checked for previous infection and experimentally inoculated with different doses of a pathogenic *Y. pestis* strain. Anti-F1 antibody titers were followed as witness of this immune response. According to the dose, mortality ranged from 18% to 37% of the rats. Up to 10^6^ bacteria can be present in the flea’s midgut [17], but the number of bacteria transmitted during a blood feeding varies from zero to 4,000 [29]. Although individual black rats in Madagascar may be infested with up to 70 fleas, our results still support previous findings suggesting that under field conditions many wild *R. rattus* may survive infection [8]. However chronic infection was not detected in survival rats, suggesting that carriage of fleas by these rats might be more important for maintenance of endemic foci than carriage of bacteria.

Regardless of the dose injected, all rodents displayed similar antibody kinetics, with anti-F1 IgM maximal at Day 13 after infection and IgG at Day 25. IgM titers reached a plateau for 500 cfu inoculated, and decay is rapid after one month [22], [30]. Individual variation exists with 10% of the rats staying IgM negative for the highest dose inoculated and 10% of rats inoculated with more than 1,500 cfu also remaining IgG negative. To date no *Y. enterocolitica*, *Y. pseudotuberculosis* or F1-negative *Y. pestis* strains, which could explain resistance of the wild rats to *Y. pestis*, were ever described in Madagascar. The survival of these rats thus suggests mechanisms of control of the bacteria other than antibodies or at least anti-F1 independent. No specific parameters of the rats correlate with this negative serology. We also found wide differences in antibody titers according to the village of sampling. In line with early reports [14], our group has already described differences in sensitivity to *Y. pestis* for rats from plague endemic and non-endemic areas of Madagascar [8]. However, differences here involved villages within the endemic area. Recently Bei Li et al suggested that between-rat differences in antibody response could be due to a limitation of the number of *Y. pestis* at early stages of infection by an efficient polymorphonuclear leukocyte and macrophage response at the infection site [31]. In our case we used subcutaneous inoculation which is unlikely to induce this clearance mechanism.

The long-term persistence of the serologic response to F1 antigen was evaluated after plague infection in a second batch of rats. Two groups could be distinguished among the rodents i) a minor group with long-lasting antibodies and ii) a major group with a rapid decay of antibody titers. There was some suggestion that rats from the long-lasting group had a lower weight and, in this experiment, weight also correlated with some other kinetic parameters. Although, this may suggest differences in long-term immune responses between rats of different age, as this study was conducted on wild rats many other factors may play a role (e.g. previous exposure to *Y. pestis* or to other pathogens). Moreover, there was no effect of weight on any immune response parameter collected during the short-term experiments.

Irrespective of whether or not rats with long-term persistence of IgG antibodies are only found within a sub-set of the population, if they are resistant to future infection there may be implications for plague transmission from one year to another. We investigated recently the role of a first inoculation on the survival of the rat after a second inoculation, and we showed that the survival increased with the dose inoculated during the first injection (Andrianaivoarimanana et al in preparation). Indeed, if a population contains high numbers of resistant rats, subsequent plague outbreaks may be less likely. Alternatively, although many infected fleas show high rates of mortality [7], the presence of resistant rats may allow the long term survival of a few infected fleas, facilitating persistence. However, some laboratory studies argue against a significant role of the persistence of IgG antibodies. In mice, several studies suggest that short term immunity could be antibody dependant whereas long term immunity may be cell mediated [32], [33], and protection against experimental challenge was found to be mainly CD4+ T cells dependent [34]. Thus, rapid increase of IgM could be an important factor of survival of rats during an outbreak of plague in villages like Ambohimasina, but the role of IgG long-lasting immunity in the maintenance of plague foci requires more investigation. Moreover, *Y. pestis* strains lacking F1 could still be virulent for these rodent populations [20], [35], [36].

Finally, we compared RDT and ELISA for the detection of anti-F1 antibodies to analyze their usefulness for epidemiological investigation. Our results indicate that a high proportion of exposed rats may remain ELISA positive for many months, and therefore detection of anti-F1 IgG in wild rat populations doesn’t necessary reveal a recent outbreak. In our previous study of the RDT, its sensitivity and specificity for rodent sera were 87.8% and 90.3% respectively [26]. However, we didn’t evaluate these parameters in relation to the antibody titer in seropads or the time course of antibody production after infection. Here, we confirmed the utility of anti-F1 dipsticks for the detection of IgG with an overall sensitivity compared to the standard ELISA of more than 80%. Thus, serological investigations of foci are useful even several months after transmission. Crucially, the RDT allows such surveys to be conducted on a range of potential reservoir species and in the absence of the specific equipment required for ELISA.

In conclusion, this study confirmed that a low numbers of bacteria can be sufficient to induce an immune response in wild black rats from Madagascar, and that survival following realistic infection doses under field conditions may be frequent. Immune responses appear quickly and a small proportion of rats can maintain this response over one year, although the significance of this for plague epidemiology is still uncertain. Interestingly, the immune response appeared to differ between villages to another, which also may have implications for plague epidemiology. More studies are needed to investigate heterogeneity in rat immune responses and the relative importance of genetic and other factors.
